# The Utility of Research Domain Criteria in Diagnosis and Management of Dual Disorders: A Mini-Review

**DOI:** 10.3389/fpsyt.2022.805163

**Published:** 2022-03-01

**Authors:** Benyamin Hakak-Zargar, Aarya Tamrakar, Tessa Voth, Armita Sheikhi, Jennifer Multani, Christian G. Schütz

**Affiliations:** ^1^Faculty of Health Sciences, Simon Fraser University, Burnaby, BC, Canada; ^2^Behavioral Reward Affect + Impulsivity Neuroscience Lab, Department of Psychiatry, Faculty of Medicine, Institute of Mental Health, University of British Columbia, Vancouver, BC, Canada; ^3^Department of Psychology, Faculty of Arts, University of British Columbia, Vancouver, BC, Canada; ^4^Department Biomedical Physiology and Kinesiology, Faculty of Science, Simon Fraser University, Burnaby, BC, Canada; ^5^School of Kinesiology and Health Science, York University, Toronto, ON, Canada

**Keywords:** Research Domain Criteria (RDoC), concurrent disorders, substance use disorder, dual disorders, psychiatric management models

## Abstract

The Research Domain Criteria (RDoC) initiative has been considered a comprehensive alternative classification framework for understanding neuropsychiatric ailments, as opposed to the longstanding, traditional DSM framework. Where the DSM categorizes neuropsychiatric disorders as each being distinct and diagnostically defined by the presence of specified symptoms, RDoC provides a multidimensional conceptualization of psychiatric disorders with neurobiological roots. By taking a multidimensional approach, RDoC overcomes two major constraints of the DSM framework: that is, that the DSM is categorical in its approach to psychiatric disorders to the point of understating the intersectionality between concomitant disorders, and that the DSM focuses mainly on clinical features. RDoC seems to better account for the intersection between dual disorders and considers a range of factors, from the more microscopic (e.g., genetics or molecular functions) to the more macroscopic (e.g., environmental influences). The multidimensional approach of RDoC is particularly appealing in the context of dual disorders. Dual disorders refers to a concurrent psychiatric disorder with an addiction disorder. RDoC accounts for the fact that there is often overlap in symptoms across and bidirectional influence between various disorders. However, to date, there is limited research into the clinical utility of RDoC, and less so in the context of the clinical management of dual disorders. In this Mini-Review, we discuss how RDoC differs from the DSM, what outcomes have been reported in utilizing RDoC clinically, the utility of RDoC for the diagnosis, management, and monitoring of psychopathology, and the limitations of RDoC as well as avenues for future research.

## Introduction

There are several approaches developed by various organizations to classify mental health disorders. The American Psychiatric Association published its 5th edition of the Diagnostic and Statistical Manual of Mental Disorder (DSM-5) in 2013, while the U.S. National Institute of Mental Health produced its Research Domain Criteria (RDoC) in 2009. While there are clear overlaps in the approaches taken by these organizations, there are some key distinctions that make each method more suitable for a different purpose (1). The DSM-5 is designed purely as a diagnostic tool and views disorders as distinctly separate. The classification of mental disorders into distinct categories follows the traditional clinical approach of identifying disorders as distinct categorical entities.

Its findings are primarily based on self-reported information from patients who often have varying levels of understanding regarding these phenomenology-based psychological symptoms, which could lead to contradictory diagnoses. The RDoC, however, has a very different approach, primarily focusing on linking neuroscience research findings to clinical phenomena and ultimately diagnoses. RDoc is an alternative approach to the DSM-5 series and is not intended to be a direct replacement (2). While the current RDoC framework shows noteworthy promise, there are challenges and concerns that need to be addressed to reach its potential for use in clinical settings (3).

Dual disorders, also known as concurrent disorders, are one of the most challenging psychiatric problems of our time. Most commonly, the term “dual disorders” is applied to indicate the diagnosis of a co-existing psychiatric disorder and substance use disorder (SUD). Dual disorders imply that patients have multiple needs, necessitating that multiple sectors within health care services are involved in providing care for dual disorder patients. However, many if not most patients with a dual disorder are not identified by health services and do not receive adequate treatment. In addition, these services are not sufficiently linked to one another in providing care, leading to fragmentation and lack of continuity of care. The risk exists that patients are shunted between services and that they eventually drop out of care. Research confirms this concern by explaining that dual disorder patients might not always meet the criteria for treatment within a specific service (mental health or substance use), and they might be referred back and forth between these services without a specific service taking responsibility for their care (4). Other studies add that even when dual diagnosis patients have access to treatment, this treatment might not be tailored to their specific needs. This raises concerns, as dual disorders are associated with a poor prognosis, complex needs, increased severity of symptoms, poor treatment adherence, and increased contact with the criminal justice system. The complexity and the increased prevalence of dual disorders necessitate the need for a comprehensive and standardized neurodiagnostic assessment.

Despite the application of RDoC in research and in the classification of mental health disorders, there is limited knowledge regarding its utility for SUD, and consequently dual disorders. We conducted a comprehensive review of the literature focusing on the published literature to the utility of RDoC in the management and diagnosis of dual disorders across clinical settings. This mini-review will provide a narrative summary of the literature, gaps, and the future direction for this emerging tool.

## RDoC vs. DSM-5

The RDoC project was initiated in 2009 by the NIMH as a response to an increased understanding of the importance of factors that were not accounted for in existing diagnostic systems (such as neurobiological systems) in understanding psychopathology (1, 5). The main goal of this new system was to incorporate research findings from various fields to create a research system that looks at multiple levels of human functioning impacted by a given pathology instead of conducting research based on the limited symptom-based diagnoses used in the DSM-5 (6–8).

An experimental approach to the new research framework was deemed necessary, given the budding state of the science of mental disorder and the constraints of research based on current classification systems. It was apparent that if developments in basic and translational science were to be applied to the science of mental disorder, a long-term approach would be needed. Such an approach would need to examine psychopathology with reference to behavioral and brain mechanisms rather than in terms of existing disorder categories (1, 2, 8). RDoC is not intended to replace the existing diagnostic systems that guided research, but rather to supplement it and to encourage research into the wider range of mechanisms that are disrupted in mental and SUD (2, 9, 10). RDoC was developed to respond to the existing reliance on the DSM-5 and ICD diagnostic categories, with the intention of guiding research and the limitations placed on research by their structures (1). The fact that new research has failed to support existing diagnostic systems in their ability to capture the full range of factors impacting psychopathology supports the use of RDoC in tandem with the DSM-5 and ICD (1, 8, 10).

## RDoC Differs From the DSM-5 in a Few Important Ways

First, it is based upon a fundamentally different approach to dimensionality, one more in line with DSM-5's Alternative Models for Personality Disorders than current diagnostic procedures are (1). RDoC considers six “domains” of human functioning and behavior (negative and positive valences, cognitive systems, systems for social processes, arousal/regulatory systems, and sensorimotor systems) that can be impacted by a particular condition. These six domains provide categories into which a variety of more specific factors, or “constructs,” can be divided. These constructs, and the domains into which they fall, can be each analyzed across a number of specific units of analysis, such as genes, molecules, cells, circuits, physiology, behavior, and self-reports (6–8). This design encourages the analysis of different facets of pathology on multiple levels, thus allowing for a better understanding of the many factors at play which might not be considered in the DSM-5. Neurobiology, in particular, is one factor that was given limited consideration in the DSM-5, but would be analyzed in the RDoC approach (1, 2). Critically, the goal with RDoC is not to explain current syndromes in terms of these dimensions; rather, it is to characterize the negative effects that result from an abnormality in a given dimension or interacting set of dimensions (1).

Second, the RDoC project was not intended for practical clinical use in the near future. Rather, it provides a framework for research. It does not formally incorporate any current ICD or DSM disorders; in fact, it does not define mental disorder or any specific disorders at all (1, 2). It is simply a research tool to facilitate more in-depth research into psychopathology, and in doing so, it avoids taking a “symptom-first” approach to psychopathology. This allows for the possibility of it being used for preventative medicine, as opposed to the curative nature of current diagnostic systems (11).

**Figure 1:**
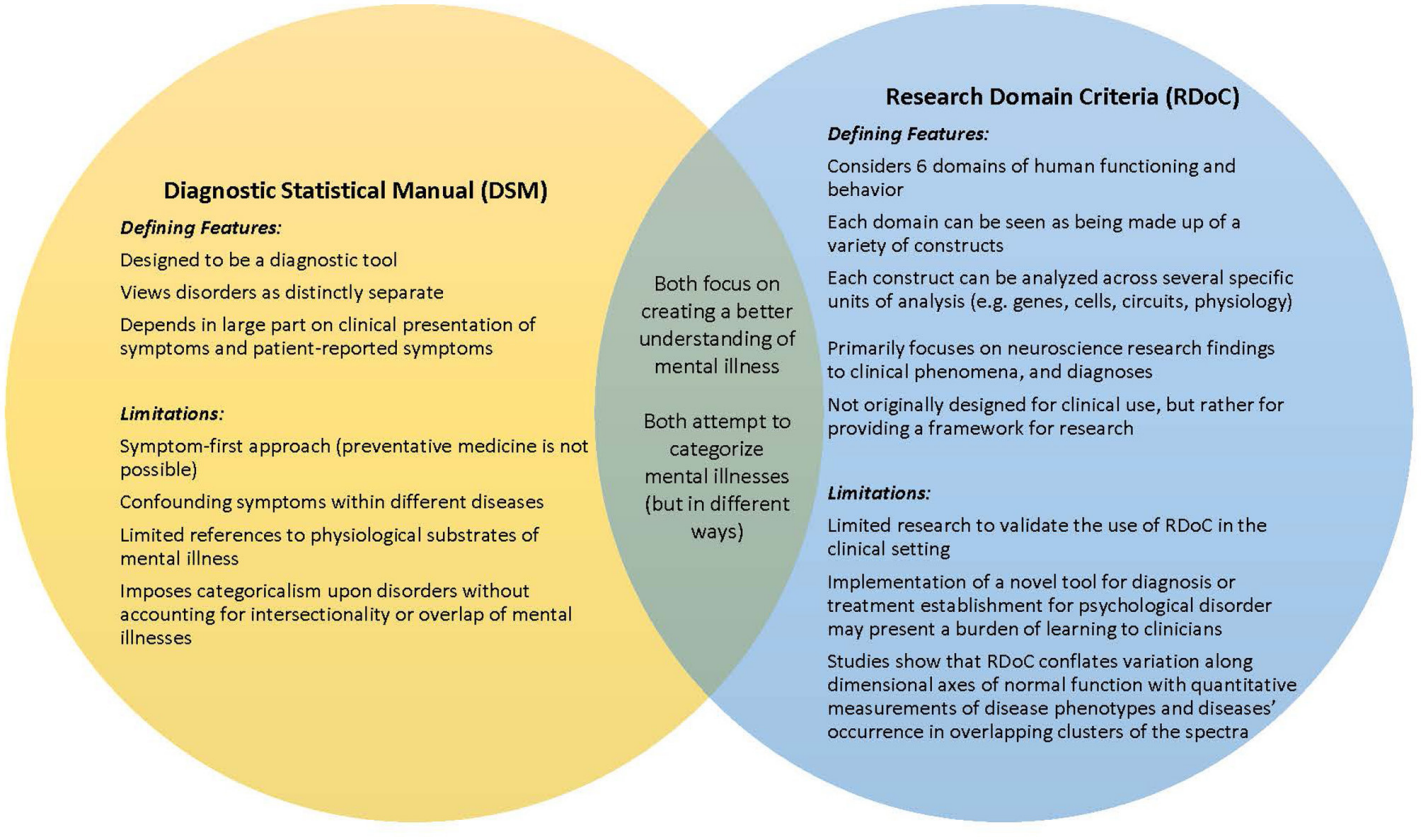
Comparison between the diagnostic statistical manual and the research domain criteria approaches to dual disorders.

Given those primary differences, in our review we will be taking a look at these two different systems in order to understand the utility of RDoC in the clinic setting. A summary of these differences have been provided in Figure 1.

## Clinical Use of RDoC

RDoC attempts to approach psychopathology with reference to neurobehavioral mechanisms rather than classify them into pre-existing categories. In principle, RDoC takes patients' cognitive, emotional, social, and behavioral experiences, or in other words, subjective experiences, into account as an equal counterpart to the brain or other biological processes (3, 12).

The primary argument being put forth here is that there is evidence of neurobiological factors at play for psychopathology, which suggests not only a means for increased understanding of mental illness, but also an avenue for management and monitoring of these illnesses by tracking and perhaps even directly addressing these same neurobiological parameters. There are a number of examples of psychopathologies that have been associated with specific neurobiological markers; four of these examples have been summarized in brief here.

As a first example, SUD, which are in part characterized by compulsivity and impulsivity, are linked to a reduction of arousal and termination of behaviors, cognitions, and affect, observable as a shift over time from ventral to dorsal striatum activation (6). Studies into genetic factors that may predispose individuals to SUD link impulsivity behaviors related to addiction with genes encoding cannabinoid brain receptor type 1 and mu-opioid receptor type 1, which both play a role in the corticolimbic reward pathway (6). Structural variation in large-scale brain systems related to motor inhibitory control, including the cortico-thalamic-striatal-cortical circuitry, may mediate a component of the genetic risk for compulsivity (6). Furthermore, there is compelling evidence that specific mutations in glutamatergic striatal kainate receptor genes are linked to perseverative and repetitive behaviors common to compulsivity and may well be a candidate biomarker for therapeutic monitoring (6).

Alcohol use disorder (AUD), a specific type of substance use disorder, has likewise been found to have a number of specific genetic neurobiological factors which contribute to the likelihood of its development (13). Broadly speaking, the likelihood of AUD developing seems to relate to an individual's subjective response to alcohol: a reduced response to the sedative and unpleasant effects increases risk of AUD, while, independently, a higher sensitivity to the stimulant and pleasant effects of alcohol likewise increases risk of AUD (13). How this subjective response manifests, both in pleasant and unpleasant ways, is highly multifactorial, influenced by both genetics/neurobiology and lifetime experiences (13, 14). Several genes have been found to play a role, including genes influencing alcohol metabolism, as well as opioidergic, dopaminergic, GABAergic, serotonergic, and neurosteroidergic genes (13, 15). Some specific examples include the ALDH2 gene, which plays a role in alcohol metabolism and has been linked to AUD, and similarly the ADH1B gene, which is involved again in alcohol metabolism and is associated with decreased sensitivity to the pleasant effects of alcohol (13). Some gene variants affecting alcohol dehydrogenase (a key enzyme in alcohol metabolism) lead to highly unpleasant effects from alcohol, such as flushing, headaches, tachycardia, and nausea (13). Genes affecting the GABA_A_ receptor, as well as genetic variation in nicotinic acetylcholine receptors and polymorphism in serotonin transporter gene SLC6A4, have been associated with attenuation of the aversive/sedative subjective responses to alcohol (13, 15). A single nucleotide polymorphism (SNP) in the mu opioid receptor gene (OPRM1), specifically the Asn40Asp SNP, seems to be a key facilitator of the stimulant and pleasant subject responses to alcohol (15). Litten et al. (14) suggest that the DSM-5 be utilized to diagnose AUD, but, given the immense variability in the presentation of this disorder and our increasing understanding of the diverse neurobiological factors at play, they suggest that an Alcohol Addiction RDoC, or AARDoC, be used subsequent to diagnosis to personalize treatment to the individual.

Another example can be seen with internet gaming disorder (IGD). This condition has been associated with higher activity in the superior medial frontal gyrus, right anterior cingulate cortex (ACC), right superior and middle frontal gyrus, the left inferior parietal lobule, the left precentral gyrus, and the left precuneus and cuneus, suggesting worse response-inhibition and impaired prefrontal cortex functioning, alongside decreased activity in the bilateral middle and inferior temporal gyri, and the right superior parietal lobule, suggesting decreased visual and auditory functioning. In the context of IGD, decreased white matter density has been demonstrated in the inferior frontal gyrus, insula, amygdala, and anterior cingulate, indicating reduced capacities for decision-making, behavioral inhibition, and emotional regulation. Studies have also found increased volume in the right caudate and nucleus accumbens (pleasure centers of the human brain) and decreased resting-state functional connectivity in the prefrontal cortex (suggesting decreased cognitive control), similar to that which is seen in SUD (8). As with SUD, IGD is associated with greater impulsivity, which has been suggested to be related to abnormalities in gray matter in areas related to executive control (e.g., decreased gray matter density in dorsomedial prefrontal cortex, the orbitofrontal cortex, bilateral insula, amygdala, and fusiform) (8). These changes relate to impaired behavior inhibition, attention, and emotional regulation, which may contribute to impulse control problems (8). It was found that brain dopamine D2 (D2)/serotonin 2A (5-HT2A) receptor function and glucose metabolism is altered in those with IGD, suggesting that individuals with IGD have significantly decreased glucose metabolism in the prefrontal, temporal, and limbic systems. It has been proposed that D2/5-HT2A receptor-mediated dysregulation of the orbitofrontal cortex in particular underlies a mechanism for loss of control and compulsive behavior in individuals with IGB (8).

For a fourth example, gambling disorder (GD) has some overlap neurobiologically with IGD, including impaired activity in the prefrontal cortex leading to reduced cognitive control (10). Diminished volume in the left hippocampus and right amygdala is also associated with GD, which in turn are associated with higher scores on the behavioral inhibition system scale (i.e., decreased tendency to avoid punishment). Reduced striatal activation is seen in GD during reward anticipation and reward outcomes, thought to be correlated with lower dopamine receptor availability in the striatum. This correlates with mood-related impulsivity and behavioral disinhibition (10). Although there are overlaps between the neurobiological markers of SUD, IGD, and GD, it is notable that diffusional kurtosis imaging has found significant differences in the microstructures of the brain associated with each of these conditions (8). Notably, these conditions are commonly comorbid with mood disorders, thus becoming dual disorders. Where the DSM-5, based upon the symptoms of the patient, may miss the presence of the comorbid condition, RDoC offers a more comprehensive diagnostic and management approach across its domains and units of analysis, so that underlying factors (such as the presence of comorbid conditions) as well as symptoms are addressed.

Crucially, it has been seen in genome-wide association studies (GWAS) that psychiatric disorders are both phenotypically and genetically highly heterogeneous, not to mention polygenic and pleiotropic (16, 17). Furthermore, not only do we see that there is overlap between disorders in their symptoms, but there is also overlap in the genetic associations seen in various disorders (17). For example, in a GWAS of cannabis dependence, it was found that there was consistent overlap in genetic patterns associated with higher risk for major depressive disorder and schizophrenia; likewise, there is overlap in the genetic risk factors for obsessive compulsive disorder and schizophrenia, and overlap between genetic risk factors for generalized anxiety disorder, bipolar disorder, and schizophrenia (17). Therefore, as we continue to increase our understanding of these neurobiological markers in the above mental health disorders as well as others, the RDoC matrix and dimensions may become all the more useful in researching and, crucially, treating and monitoring not merely the symptomatic and behavioral aspects of these disorders, but the underlying neurobiological and genetic contributors, while taking into account that each psychiatric disorder may not be completely phenotypically or genetically distinct. That said, even though RDoC in theory offers a less restrictive approach and opens doors to a more well-rounded classification system, its clinical utility requires greater research. Work is currently being done in this area; below, we discuss three specific psychopathologies that have been studied for diagnosis using RDoC.

Firstly, RDoC's clinical utility in the use of antidepressants has been studied, and is supported, but the evidence casts a question of discriminant validity between some of the constructs in the classification system. To put it more specifically, potential threat and loss are separated as two distinct constructs, but there is no evidence that they should not be combined into one (7). In the domain of cognitive systems, RDoC may provide a broader and transdiagnostic approach to understanding suicidal behavior, which may lead to better suicide prevention and treatment models (5).

As a second example, in a study examining temper loss in children, RDoc was found to perform better than DSM-5 criteria in predicting the development of mood or disruptive disorders (18). Notably, using the RDoC framework, the risk of developing a disorder was found to be significantly elevated, as much as 67%, at levels of temper loss that are considered normative by current criteria (18). These findings highlight the advantages of using a truly dimensional framework, like RDoC, as opposed to the more traditional categorical approach.

As a third example, a study identified three neurobiologically unique psychosis subtypes which do not follow traditional diagnostic boundaries (19). Even though these subtypes have unique underlying structures, there was a significant overlap in the behavioral symptoms displayed by each subtype, indicating that behavior can have multiple biological causes. In this reality, using a biologically driven nosology such as RDoC has the potential to drastically increase not only the reliability but the validity of the clinical diagnosis.

While these studies prove promising, their impact is not likely to be felt by practicing clinicians for some time. A more immediate issue of adopting a new nosology system facing clinical researchers is the compatibility between old and new diagnostic systems. If the two nosologies are too discrepant, clinicians and clinical researchers would have to learn an entirely new vocabulary to discuss cases.

Through all the domains that have been examined, there is unity in suggesting that more research is needed to increase RDoC's utility in having a more comprehensive assessment of psychopathology (5).

## Limitations of RDoC

Research indicates that the RDoC paradigm may be valuable for understanding normal human psychology with conditions interpreted as extremes of normal variation. Further, studies show that RDoC conflates variation along dimensional axes of normal function with quantitative measurements of disease phenotypes and diseases' occurrence in overlapping clusters of the spectra. This moves away from the medical model of mental illnesses. RDoC contrasts with our current classification systems, the DSM-5, which defines psychiatric disorders based on clusters of symptoms instead of constructs derived from neurobiological mechanisms. In addition, RDoC aims to eliminate the normal to abnormal dimensions of these so-called fundamental behavioral concepts, which then can overlap and interact to constitute, perhaps, new clusters of symptomatology. Cuthbert and Insel (20) pointed out that taking a dimensional approach also allows for non-linear patterns to emerge. Limitations to further study of RDoC will be to design valid measures capable of capturing the full range of these dimensions, with appropriate sensitivity to transitional junctions along these dimensions associated with impaired functioning. However, in its current form, the RDoC may be a limited theoretical model intended to provide a complete understanding of why mental illness develops, how it progresses, and how different treatments might control it. Previous studies raise the question of whether a more comprehensive version of the RDoC or a different paradigm altogether by incorporating diagnostic assessments, the DSM-5 and RDoC, will be needed to guide clinical research and clinical practice in psychiatry.

## Discussion/Conclusion

Dual disorders refers to a concurrent psychiatric disorder (e.g., depression, anxiety, schizophrenia) with an addiction disorder. RDoC allows a more intersectional approach to concurrent diagnoses, while the DSM is argued by some to be the stronger framework in a clinical setting, where the distinction between disorders enables a more clear-cut diagnosis. That said, the fact is that there is considerable overlap in symptoms across various disorders, and in the event of dual disorders, bidirectional influence between the disorders can usually be expected. Moreover, our understanding of the neurobiological markers for psychopathologies is continuing to grow, providing an opportunity for disease management that must not be neglected. In that light, a better understanding of transdiagnostic concepts is desirable. To that end, we explored through a mini-review how RDoC provides a greater multidimensional and transdiagnostic understanding of dual disorders, and how RDoC has already shown promise in clinical use. That being said, the overall utilization and study of RDoC in dual disorder is still in its early stages and more research in the field is warranted. Basic research is still needed to better understand the interconnection between the neurobiology of psychological disorders (including dual disorders) and their forms, onset, course, and sociocultural processes; this will then support ongoing research into the integration of RDoC into clinical practice. Alongside this, extensive research will be needed to establish the validity of clinical use of RDoC for each disorder. This will likely demand novel research strategies to better analyze how multiple factors simultaneously and interactively impact psychopathology (1). Ideally, future studies will not only incorporate a multidimensional view of the various factors that intersect in psychopathology, but will also take a transdiagnostic approach, taking into consideration how dual (or multiple) disorders interact, overlap, and, crucially, may be treated intersectionally.

## Author Contributions

BH-Z supervised the project, conducted the literature review, and drafted and edited the manuscript. AT and JM conceptualized the research topic, conducted the literature review, and drafted the manuscript. TV drafted and edited the manuscript and conducted the revisions. AS drafted the manuscript. CS supervised the project and reviewed and edited the manuscript. All authors contributed to the article and approved the submitted version.

## Conflict of Interest

The authors declare that the research was conducted in the absence of any commercial or financial relationships that could be construed as a potential conflict of interest.

## Publisher's Note

All claims expressed in this article are solely those of the authors and do not necessarily represent those of their affiliated organizations, or those of the publisher, the editors and the reviewers. Any product that may be evaluated in this article, or claim that may be made by its manufacturer, is not guaranteed or endorsed by the publisher.
